# Eco-Friendly Removal and IoT-Based Monitoring of CO_2_ Emissions Released from Gasoline Engines Using a Novel Compact Nomex/Activated Carbon Sandwich Filter

**DOI:** 10.3390/polym17111447

**Published:** 2025-05-23

**Authors:** Saad S. M. Hassan, Nora R. G. Mohamed, Mohamed M. A. Saad, Yasser H. Ibrahim, Alia A. Elshakour, Mahmoud Abdelwahab Fathy

**Affiliations:** 1 Department of Chemistry, Faculty of Science, Ain Shams University, Abbasia, Cairo 11566, Egypt; 2 Air Pollution Research Department, Environmental Research Division, National Research Centre, Giza 12622, Egypt; 3 Spinning and Weaving Engineering Department, Textile Industries Research Institute, National Research Centre, Giza 12622, Egypt

**Keywords:** removal of carbon dioxide, gasoline combustion, nonwoven fabric filters, polyamide (Nomex) fabrics, gas filtration efficiency, activated carbon (AC)

## Abstract

A novel cost-effective, rapid, and eco-friendly method was described for the removal of carbon dioxide (CO_2_) from the gaseous emissions of gasoline engines. This involved the use of a sandwich filter (~10 cm diameter) made of a nonwoven poly (*m*-phenylene isophthalamide) (Nomex) fabric loaded with a thin layer of activated carbon. The optimized filter, with an activated carbon mass of 2.89 mg/cm^2^, a thickness of 4.8 mm, and an air permeability of 0.5 cm^3^/cm^2^/s, was tested. A simple homemade sampling device equipped with solid-state electrochemical sensors to monitor the concentration levels of CO_2_ before and after filtration of the emissions was utilized. The data were transmitted via a General Packet Radio Service (GPRS) link to an Internet of Things (IoT)-based gas monitoring system for remote management, and real-time data visualization. The proposed device achieved a 70 ± 3.4% CO_2_-removal efficiency within 7 min of operation. Characterization of the filter was conducted using a high-resolution scanning electron microscopy (SEM), energy dispersive X-ray analysis (EDX) and Brunauer–Emmett–Teller (BET) analysis. The effects of loaded activated carbon mass, fabric type, filter porosity, gaseous removal time, and adsorption kinetics were also examined. The proposed filter displayed several advantages, including simplicity, compactness, dry design, ease of regeneration, scalability, durability, low cost, and good efficiency. Heat resistance, fire retardancy, mechanical stability, and the ability to remove other gasoline combustion products such as CO, SO_x_, NO_x_, VOCs, and particulates were also offered. The filtration system enabled both in situ and on-line CO_2_ real-time continuous emission monitoring.

## 1. Introduction

Carbon dioxide is a major component of the greenhouse gas family (GHG), emitted by human activities. It contributes more than 60% of global warming due to its increasing concentration in the atmosphere due to burning fossil fuels in industrial sites and in engines used to generate energy [1]. The increase in CO_2_ emissions due to the large consumption of fossil fuels has put the world at great risk if emissions continue to accumulate unabated in the atmosphere. This accumulation disturbs the global carbon cycle, leading to a global warming impact and climate change [2]. The effects of global warming range from ecological and physical to health impacts, including extreme weather events such as storms, crop growth, floods, heat waves, sea-level rise, and disrupted water systems [3,4]. Nowadays, many parts of the world severely suffer from all of these problems and need an urgent solution to stop the increase in atmospheric greenhouse gas (GHG) levels and their devastating impacts on climate change. Carbon dioxide removal (CDR) is now recognized as a critical component for achieving ambitious climate goals [5,6].

However, over the past two centuries, humankind has increased the concentration of CO_2_ in the atmosphere from 280 to more than 380 parts per million by volume, and it is still increasing every day [5]. Global carbon dioxide emissions increased from 0 in the year 1850 to 32,500 million metric tons in 2010, and they are expected to reach 39,000 million metric tons by 2030 [7]. According to the statistics, almost a quarter of carbon emissions are produced by the transportation sector due to emissions from combustion engines [8]. The international treaties concerned by legally binding targets to cut emissions of carbon dioxide and other greenhouse gases are those in the “Kyoto Protocol of 1997” [9] and the “Paris Agreement of 2016” [10]. According to the Intergovernmental Panel on Climate Change (IPCC), GHG emissions must be reduced by 50 to 80 percent by 2050 to avoid or reduce the dramatic dangerous consequences [11].

These treaties create a strong interest and stimulate worldwide efforts to control and reduce the emission of carbon dioxide from their sources. Many methods have been developed for capturing (CDC) and reducing or removing (CDR) carbon dioxide to meet the global goal established by these treaties and other UN regulations. Various treatment methods have been suggested for mitigation, including adsorption, absorption, condensation, membrane processes, cryogenic distillation, and catalytic oxidation. The advantages and limitations of these methods have been reviewed [12,13,14]. However, amine liquid scrubbing technology is the most widely used technique for removing CO_2_ from flue gases and industrial gas streams [15,16,17]. This technique involves the use of columns containing hazardous corrosive chemicals and requires continuous absorbent regeneration [18].

The dispersion and grafting of organic amines onto solid supports such as activated carbon, zeolites, polymeric resins, and metal–organic frameworks (MOFs) have shown a promising CO_2_ sorption performance due to the formation of carbamate or bicarbonate complexes with CO_2_ [19,20]. Moreover, recent studies have demonstrated that amine-functionalized polymers and amide-containing materials, including those based on cellulose and other porous supports, provide enhanced chemisorption efficiency and regeneration potential [21,22]. Given that Nomex is a poly (*m*-phenylene isophthalamide), its inherent amide groups may contribute to CO_2_ affinity through similar interactions.

The present investigation is undertaken to develop, examine, and evaluate the viability and effectiveness of a novel, simple, and dry system for CO_2_ removal from gasoline engine emissions. This study introduces a uniquely designed device composed of a nonwoven sandwich fabric structure made of poly (*m*-phenylene isophthalamide) (Nomex) combined with a thin activated carbon (AC) layer. Unlike conventional approaches, our design integrates a solid-phase amine-rich polymer with activated carbon, leveraging both physical adsorption and chemisorption mechanisms for enhanced CO_2_ capture. The Nomex component, containing approximately 12% nitrogen from secondary amine functional groups, interacts synergistically with AC to target acidic gaseous pollutants. This filter material is unique due to its mechanical robustness, thermal stability, flexibility, compactness, scalability, eco-friendliness, and cost-effectiveness, making it suitable for use under harsh operational conditions. Notably, the system enables both in situ and on-line CO_2_ monitoring, a practical advantage over conventional methods. These features mark a significant advancement compared to traditional amine scrubbing and adsorption-based techniques, highlighting the novelty and application potential of this study.

## 2. Experimental

### 2.1. Materials and Instruments

**Figure 1:**
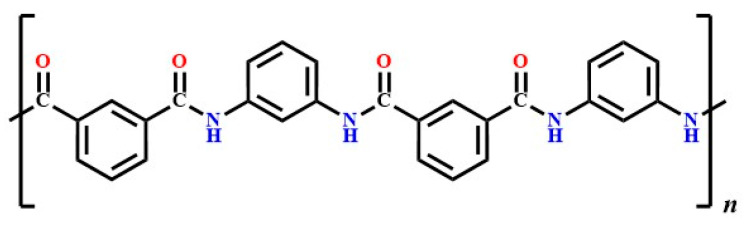
Structure of Nomex (poly (*m*-phenylene isophthalamide)).

Poly (*m*-phenylene isophthalamide) (Nomex)-based nonwoven fabrics were obtained from DuPont (Wilmington, DE, USA) through Egypt-Tex Company (Cairo, Egypt) and were characterized in accordance with international standards. The molecular structure of Nomex, shown in Figure 1, illustrated its composition as poly (*m*-phenylene isophthalamide), which contributes to its exceptional thermal stability and mechanical properties. The mass per unit area (weight) of the textile fabric materials was measured using the Standard Test Method ASTM D3776/D3776M-20 [23]. Fabric thickness was evaluated according to the Standard Test Method ASTM D1777-96(2019) [24]. The air permeability of the fabric was determined using the Standard Test Method ASTM D737-04 [25] with a Toyoseiki (JIKA) air permeability tester (Toyoseiki Co., Ltd., Tokyo, Japan). The general characteristics of the used Nomex fabric were as follows: weight: 926–1083 g/m^2^, thickness: 4.40–4.80 mm, and air permeability: 0.498–7.680 L/m^2^.s.

Activated carbon (AC), obtained from Laboratories Rasayan (LR) (Mumbai, Maharashtra, India), preheated at 60 °C for 24 h and stored in a desiccator until use. The activated carbon used had the following characteristics: particle size < 300 mesh (~50 μm), greater than 60%; acid-soluble fraction: 2.5%; water-soluble fraction: 1.5%; moisture content: 5%; ash content: 2.5%; pH value: ranging between 6.0 and 7.5; methylene blue adsorption using a 0.15% solution: more than 18 mL/0.1 g.

Scanning electron microscopy–energy dispersive X-ray analysis (HSEM-EDX) of the treated fabric was conducted using a high-resolution scanning electron microscope (SEM Quanta FEG 250 with a field emission gun, FEI Company, Hillsboro, OR, USA). Additionally, porosity and surface area assessments of the fabric were carried out using Brunauer–Emmett–Teller (BET) analysis by adsorption and desorption of nitrogen gas using a BELSORP MINI X automatic surface and pore size analyzer (MicrotracBEL Corp., Osaka, Japan).

### 2.2. Preparation of Nomex/Activated Carbon Filters

**Figure 2:**
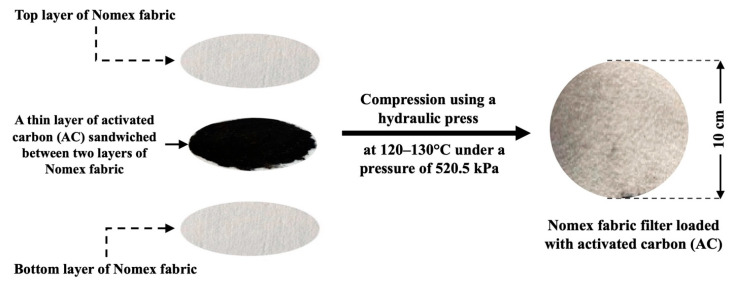
Schematic diagram of the preparation steps for the Nomex/activated carbon sandwich filter.

The preparation process of the Nomex/AC filters is illustrated in Figure 2. Different masses of activated carbon (250, 500, 750, and 1000 mg) were evenly distributed between two layers of nonwoven Nomex fabrics, each with a diameter of 10 cm. The sandwich filter was heated for 15 min at a temperature of 120–130 °C and a pressure of 520.5 kPa using a hydraulic press.

### 2.3. Equipment for Sampling and Measuring Combustion Emissions

**Figure 3:**
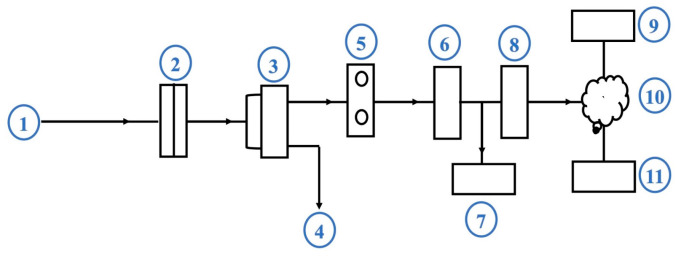
Schematic diagram of the apparatus for sampling and measuring combustion emissions. Components: (1) gas inlet; (2) filter sample holder; (3) differential pressure gauge/vacuum pump; (4) gas outlet; (5) CO_2_ sensor module; (6) sensor interface; (7) digital detector; (8) gateway (Ethernet/GPRS/WIFI); (9) local server; (10) cloud; (11) mobile phone.

A low-cost, homemade apparatus with a Nomex/AC filter was designed to evaluate the filtration efficiency. The device was assembled and used for the sampling and evaluation of the gas emissions. The apparatus consisted of an air sampler unit connected to a suction pump and a pressure meter to maintain a consistent gas flow rate of 10 L/min during the sampling process. The examined sandwich filter was placed in the filter-holder located in the vacuum chamber, which received combustion exhaust gases emitted from the generator muffler. The apparatus components are shown schematically in Figure 3.

### 2.4. Emissions Monitoring

The concentrations of CO_2_ gas passing from the emission source through the filter were monitored using solid-state potentiometric and electrical–thermal gas sensors (Alphasense Ltd., Great Notley, UK), with a measurement range of 0 to 2000 ppm for CO_2_. These sensors were integrated into a custom gas monitoring system (GMS) built on an Internet of Things (IoT) cloud platform. The GMS was fully embedded within the filter apparatus to allow the seamless, real-time tracking of CO_2_ concentrations across the filtration process.

Data collected from the sensors were transmitted wirelessly via a General Packet Radio Service (GPRS) link using the Global System for Mobile Communications (GSM) network. Secure transmission was ensured using HTTPS POST requests and validated with security keys. The validated data were stored in a cloud-based database, allowing for remote access, processing, and visualization through mobile applications. The scalability of the IoT platform enabled efficient handling for large datasets and extended monitoring sessions. These measures were implemented to ensure cybersecurity and data integrity of the transmitted data, which is particularly critical for reliable operation in mobile and vehicular applications.

### 2.5. Adsorption Technique

The adsorption capabilities and removal effectiveness of the proposed sandwich filter were investigated at the adsorption equilibrium conditions. The concentrations of CO_2_ emissions before and after filtration were measured using a gas monitoring system (GMS) connected to a cloud-based Internet of Things (IoT) platform. The concentrations of CO_2_ gas passing from the emission source through the sandwich Nomex filter (10 cm diameter) containing a thin layer of activated carbon (1.0 g) as an adsorbent were measured. After reaching the equilibrium state, the adsorption capacity (mg/g) and removal efficiency (%) of the adsorbent were estimated using Equations (1) and (2) [17,26]:

*Q_e_* = (*C_o_* − *C_e_*) × *V*/*M* (1)

Removal efficiency of CO_2_ (%) = [(*C_o_* − *C_e_*)/*C_o_*] × 100 (2)

where *C_o_* was the initial concentration of the gas pollutant (ppm), *C_e_* the concentration of the gas pollutant at equilibrium (ppm), *V* flow rate of gas emission (L/min) and *M* the mass of activated carbon adsorbent (g).

### 2.6. Kinetic Modeling

The prepared Nomex/AC filter was exposed to CO_2_ emissions from a gasoline engine at different time intervals, and the kinetic studies of CO_2_ gas adsorption were conducted. The adsorption rates were analyzed and compared with pseudo-first-order and pseudo-second-order models, as described by Equations (3) and (4), respectively [27,28].

log(*q_e_* − *q_t_*) = log(*q_e_*) − *k*_1_
*t*/(2.303) (3)

*t*/*q_t_* = 1/(*k*_2_ *q_e_*^2^) + *t*/*q_e_* (4)

where *q_e_* and *q_t_* are the adsorption capacities (mg·g^−1^) of the adsorbent at equilibrium and at any time, respectively. *t* represents the contact time (min), while *k*_1_ (min^−1^) and *k*_2_ (g·mg^−1^·min^−1^) are the rate constants of the pseudo-first-order and pseudo-second-order adsorption models, respectively.

## 3. Results and Discussion

Different sandwich filters consisting of two Nomex layers containing activated carbon in-between were tested for the removal of CO_2_ from the emissions of gasoline combustion engines. Filters with a surface area of 78.54 cm^2^, loaded with 250, 500, 750, and 1000 mg of activated carbon per 78.54 cm^2^ were prepared, characterized, and tested.

### 3.1. Scanning Electron Microscopy (SEM) and Energy Dispersive X-Ray (EDX)

The scanning electron microscopy–energy dispersive X-ray analysis (SEM-EDX) technique was used to provide a non-destructive determination of the elemental identification and quantitative composition of the adsorbent sample. EDX analysis was used to confirm the existence of the elements in the engine exhaust, the adsorbed CO_2_, and other species from the engine exhaust on the filter. EDX spectra (elemental mapping patterns) of the Nomex fabric before and after the addition of activated carbon were measured and re-examined after passing the emissions from the gasoline engines.

**Figure 4:**
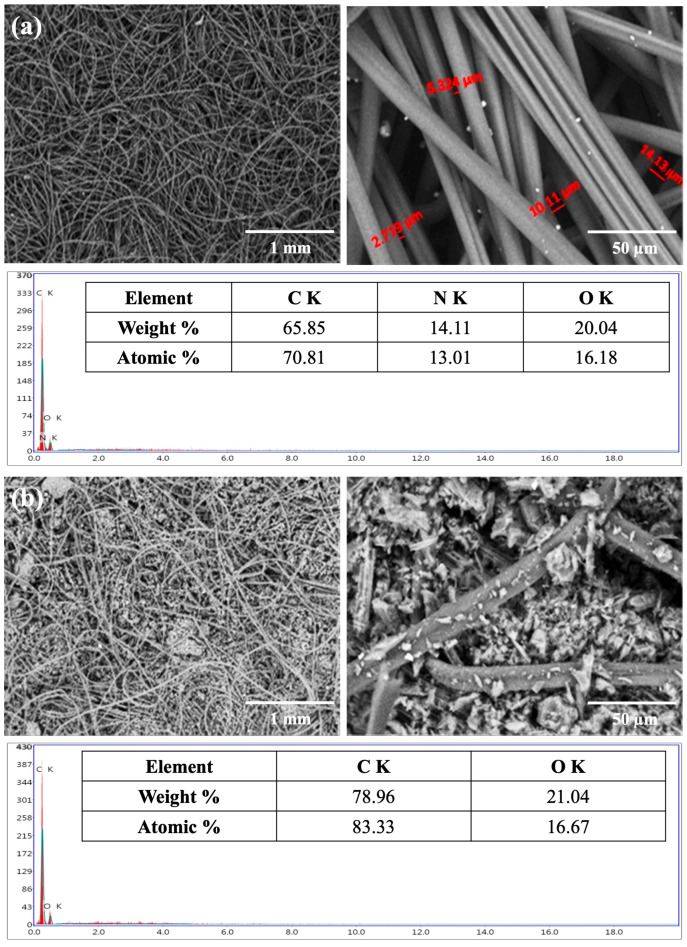
SEM and EDX analyses of Nomex filters: (**a**) blank filter before loading with activated carbon, and (**b**) filter loaded with activated carbon prior to emission filtration.

The morphology of the nonwoven fabrics before the addition of activated carbon displayed a smooth fiber surface with a normal structure (Figure 4a). The morphological structure of the filter fabric after the addition of activated carbon showed a uniform distribution of activated carbon granules within all parts of the fabric filter (Figure 4b). However, a few irregular activated carbon granules were detected on the filter surface after filtration probably due to agglomerated carbon particles attached to the fibers during the filtration process.

EDX elemental examination revealed that the dominant elemental composition of the surface of the Nomex fabric (the blank) before the addition of activated carbon (Figure 4a) was carbon (70.81%), nitrogen (13.01%), and oxygen (16.18%), which was close to the constituents of Nomex: carbon (70.58%), nitrogen (11.76%), and oxygen (13.44%). After the addition of activated carbon to the Nomex filter, EDX showed (Figure 4b) the presence of only carbon with a higher percentage (83.33%) and oxygen (16.67%). The absence of nitrogen was attributed to the complete and homogeneous coverage of the fabric surface of the filter with the activated carbon.

**Figure 5:**
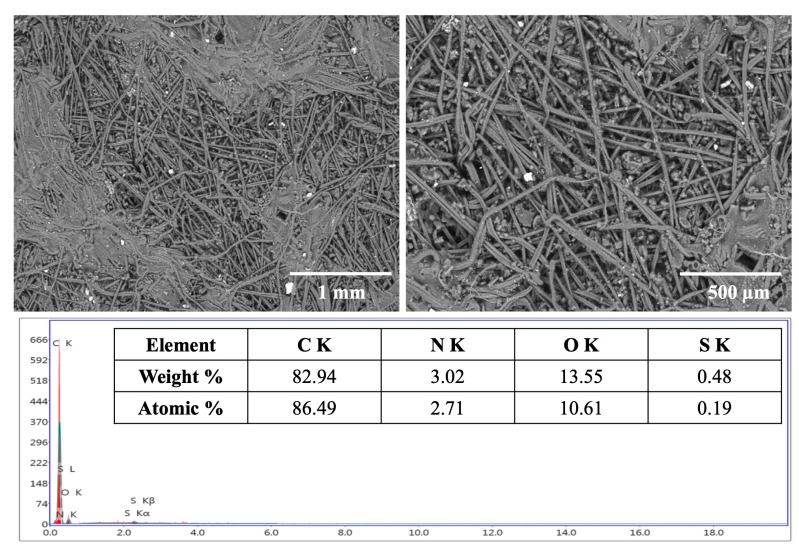
SEM and EDX analyses of Nomex filters after being loaded with activated carbon and following emission filtration.

Comparing the surface composition of the sandwich filter before and after CO_2_ filtration revealed an increase in carbon and the appearance of nitrogen and sulfur peaks. EDX showed (Figure 5) the presence of carbon (86.49%), oxygen (10.61%), nitrogen (2.71%), and sulfur (0.19%). Nitrogen and sulfur originated from nitrogen and sulfur oxides released during gasoline combustion and their subsequent retention on the carbon filter. The white patches visible in the SEM image are attributed to these retained combustion products, particularly NO_x_ and SO_x_ species, as well as possible particulate matter that agglomerates on the filter surface. These species tend to form localized accumulations at high-adsorption sites, appearing as bright regions due to their different atomic contrast in SEM imaging. The accompanying EDX spectrum and composition table confirm this interpretation, and the presence of nitrogen and sulfur, not originally detected in the pre-filtration filter, further supports their origin from engine exhaust gases.

### 3.2. Surface Area and Pore Size Distribution of the Filter

**Figure 6:**
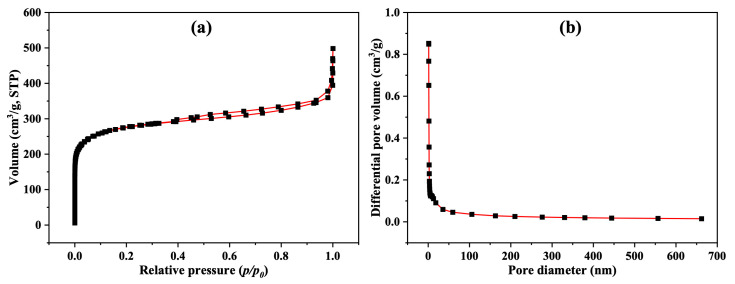
(**a**) N_2_ adsorption–desorption isotherms of AC; (**b**) the corresponding pore size distribution.

The Brunauer–Emmett–Teller (BET) method [29,30] was used to measure the surface area and porosity of the activated carbon (AC) sample used in the filter. Figure 6a,b depict the N_2_ adsorption–desorption isotherm and the pore size distribution of the sample, respectively. As shown in Figure 4a, the isotherm of the prepared sample displayed a hysteresis loop at relative pressures between 0.005 and 0.99, caused by the adsorption and desorption of N_2_ during the capillary condensation. According to the IUPAC classification system for adsorption isotherms, Figure 6a reveals a type-II isotherm, indicating that nanopores or macropores were filled with N_2_ gas to form a monolayer, followed by multilayer adsorption [31]. The measured surface area, total pore volume, and average pore diameter of the AC sample were 1010.5 m^2^/g, 0.5834 cm^3^/g, and 2.3096 nm, respectively.

### 3.3. Adsorption Kinetics

**Figure 7:**
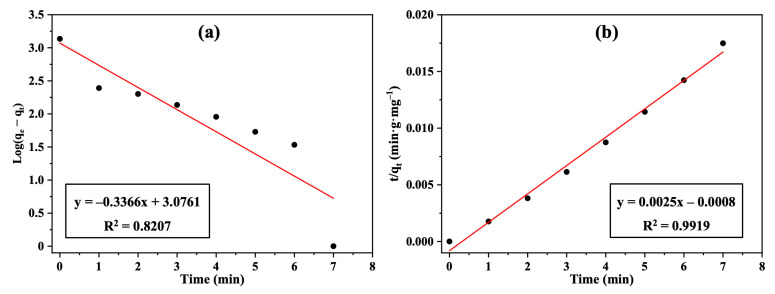
Plots of (**a**) pseudo-first-order kinetics, and (**b**) pseudo-second-order kinetics for CO_2_ removal using the Nomex/AC filter.

The relationship between the adsorbed carbon dioxide concentration and the mass of activated carbon adsorbent as a function of contact time was investigated. It was observed that the removal of CO_2_ gas increased gradually with time and reached a state of equilibrium after 7 min with a 70% removal efficiency. As illustrated in Figure 7a,b, pseudo-first-order and pseudo-second-order models were used to fit the obtained practical kinetics data. Since the pseudo-second order model displayed a higher correlation coefficient (R^2^ = 0.9919) than the pseudo-first-order model (R^2^ = 0.8207), and due to closer approximations between the practical and calculated data (*q_e_* and *q_e_^exp^*) (Table 1), it was concluded that the current adsorption process followed the second-order model. Since the present study primarily focused on adsorption kinetics and filter performance under ambient conditions, thermodynamic parameters such as the heat of sorption would not add practical useful information and were not determined.

### 3.4. Effect of Fabric Type

Fabrics made of polyester and polyamide (Nomex), with and without activated carbon (AC), were examined and compared for the removal of carbon dioxide as well as other fuel combustion products (e.g., carbon monoxide, sulfur dioxide, and nitrogen oxides). The results revealed that Nomex fabric without AC exhibited a noticeable affinity for removing acidic gases (CO_2_, SO_2_, and NO_x_), likely due to its basic nature, as well as for removing neutral gases (CO, VOC) owing to its inherent adsorption capacity. This affinity was significantly enhanced in the presence of AC, showing a synergistic effect. Furthermore, Nomex fabric demonstrated some significant advantages over polyester fabric, such as thermal stability, resistance to shrinkage and stretching, and superior overall efficiency.

The adsorption capacity observed in the Nomex/AC filter was attributed to both physical and chemical characteristics. The activated carbon provided a high surface area and porous structure, as confirmed by BET analysis, which promoted effective physisorption of CO_2_ molecules. Additionally, the Nomex fabric itself contained nitrogen-based functional groups, which facilitated chemical interactions with acidic gas species such as CO_2_. This combination of microporosity and chemical reactivity enhanced the overall adsorption performance of the filter.

### 3.5. Effect of the Mass of Activated Carbon (AC)

**Figure 8:**
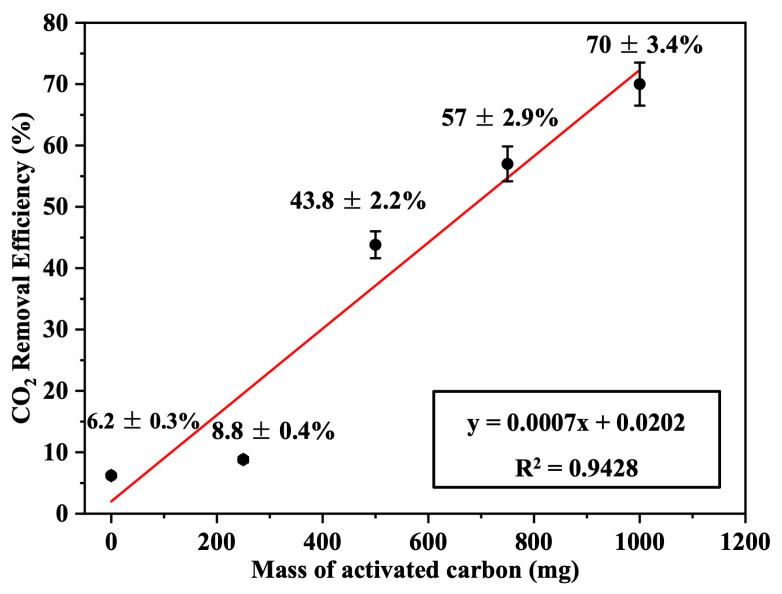
CO_2_-removal efficiency as a function of the loaded mass of activated carbon (AC) in the Nomex/AC filter (*n* = 3, error bars represent standard deviation).

The effect of the mass of activated carbon (mg) per filter unit area (cm^2^) was tested. Four Nomex filters, each with a diameter of 10 cm, were loaded with 250, 500, 750, and 1000 mg of activated carbon (corresponding to 0.72, 1.44, 2.17, and 2.89 mg/cm^2^ of carbon per fabric area, respectively) and used for CO_2_ filtration. The emission removal efficiencies were 8.8 ± 0.4%, 43.8 ± 2.2%, 57 ± 2.9%, and 70 ± 3.4%, respectively, based on triplicate measurements (*n* = 3), as shown in Figure 8. This improvement in efficiency is attributed to the increased specific filtration surface area (SFSA) of the adsorbing material, which enhances the filtration efficiency.

These results were compared with CO_2_-removal efficiencies obtained by activated carbon-based filters reported in the literature under practical or semi-commercial conditions. Although detailed specifications from commercial manufacturers are often unavailable, previous studies provide relevant performance benchmarks. For example, Maniarasu et al. [32] reported a maximum CO_2_-removal efficiency of approximately 50% using activated carbon derived from coconut shell in a compression ignition (CI) engine exhaust system. Similarly, Chue et al. [33] examined CO_2_ recovery from flue gas using commercial BPL activated carbon in a pressure swing adsorption (PSA) system and reported removal efficiencies around 53%. These systems were generally operated with larger filter masses or more complex configurations than those evaluated in the present study. In contrast, our compact, dry-type Nomex/AC sandwich filter achieved a higher removal efficiency of 70 ± 3.4% using only 2.89 mg/cm^2^ of activated carbon under ambient conditions. This highlights the material synergy, optimized structure, and enhanced adsorption efficiency of the proposed filter, suggesting practical advantages over comparable systems in terms of both performance and adsorbent utilization.

### 3.6. Effect of Filter Thickness

**Figure 9:**
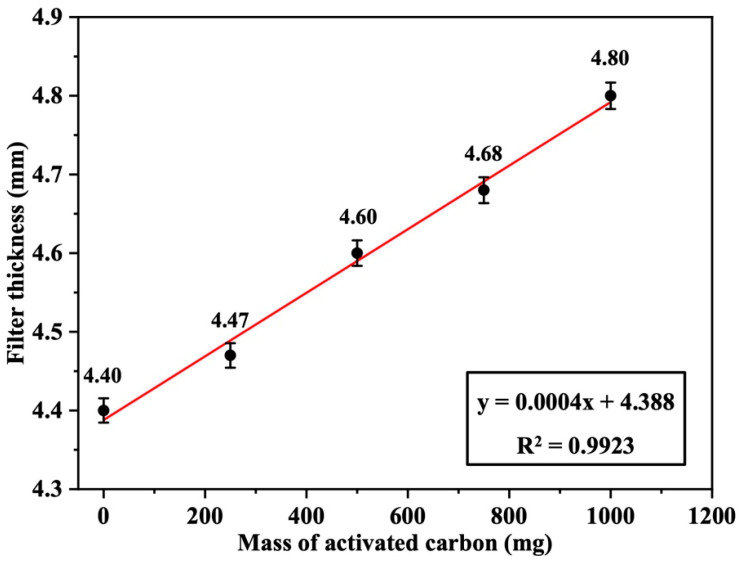
Filter thickness as a function of the loaded mass of activated carbon (AC) (*n* = 3, error bars represent standard deviation).

The thickness of the Nomex filters loaded with 250, 500, 750, and 1000 mg of activated carbon was measured and found to be 4.47 ± 0.02, 4.60 ± 0.02, 4.68 ± 0.02, and 4.80 ± 0.02 mm, respectively, based on triplicate measurements (*n* = 3). The thickness of the blank filter (0 mg carbon) was 4.40 mm. Figure 9 displays a linear increase in the filter thickness as a function of the loaded activated carbon mass, with a good positive correlation (R^2^ = 0.9923). This indicates a uniform and homogeneous distribution of activated carbon on the surface of the fabric filter under the applied temperature and pressure used during the preparation process.

### 3.7. Effect of Filter Air Permeability

**Figure 10:**
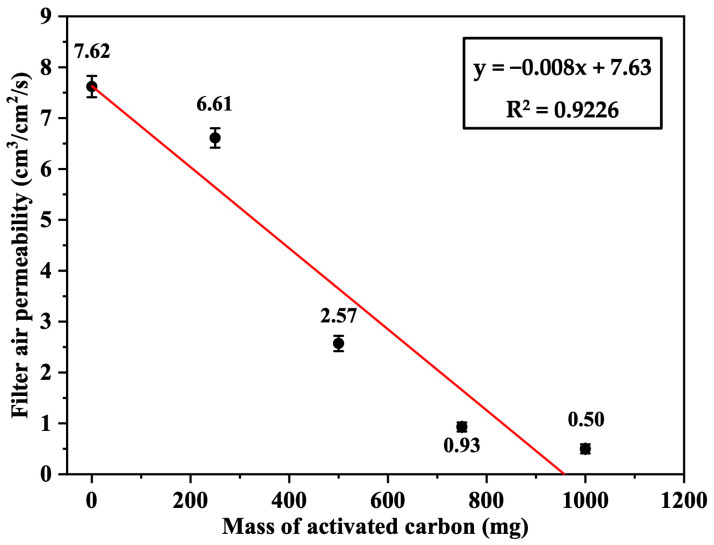
Air permeability of the Nomex/AC filter as a function of the loaded mass of activated carbon (AC) (*n* = 3, error bars represent standard deviation).

The air permeability of the Nomex sandwich filters loaded with 0, 250, 500, 750, and 1000 mg of activated carbon per filter was measured and found to be 7.62 ± 0.21, 6.61 ± 0.19, 2.57 ± 0.15, 0.93 ± 0.09, and 0.50 ± 0.08 cm^3^/cm^2^/s, respectively, based on triplicate measurements (*n* = 3). As shown in Figure 10, this demonstrated a linear decrease in filter air permeability as the mass of activated carbon increased. The negative correlation was high (R^2^ = 0.9226) and probably attributed to the high density of carbon, which reduced the airflow across the filter fabric.

### 3.8. Effect of Time and Regeneration on CO_2_ Removal Efficiency

The removal efficiency of CO_2_ as a function of time over a 15 min period was examined using the Internet of Things (IoT)-based gas monitoring system described in Section 2.4. Engine emissions were passed through the proposed Nomex/AC filter loaded with 1.0 g of activated carbon, and the average concentration of CO_2_ (mg/m^3^) was continuously monitored in real time using an embedded solid-state CO_2_ gas sensor.

The system transmitted data every 5 s to a secured cloud server, enabling the real-time monitoring and analysis of filter performance. This monitoring system allowed for immediate feedback during operation, making it highly suitable for adaptive control and practical deployment in mobile or remote environments. Sensor calibration was performed using certified CO_2_ gas mixtures to ensure accurate and reliable readings within the 0–2000 ppm range. The results showed that CO_2_ concentration reached a steady state after 7 min, with a removal efficiency of 70 ± 3.4%. These findings confirm the effective integration and real-time functionality of the IoT-based system for continuous and accurate emission monitoring.

To evaluate the operational stability and regeneration potential of the Nomex/AC filter under conditions simulating prolonged exposure to gasoline combustion emissions, seven consecutive CO_2_ adsorption–desorption cycles were conducted. Each cycle involved 15 min of exposure to engine exhaust, followed by thermal regeneration at 120 °C for 1 h. The filter maintained a consistent CO_2_-removal efficiency of approximately 70 ± 3.4% throughout the first four cycles (equivalent to 60 min of total filtration time), after which a gradual performance deterioration was observed. This behavior suggested partial saturation of the adsorption sites or the irreversible binding of combustion byproducts over time. Although the elevated regeneration temperature restores short-term performance, it may not fully desorb all of the retained CO_2_ under repeated thermal and chemical stress. Overall, the filter demonstrated stable performance under continuous operation for at least four successive cycles, confirming its durability and reusability for practical emission control applications.

### 3.9. Comparison of Some Methods Used for CO_2_ Emission Removal

A comparison of the general characteristics of some previously suggested CO_2_-removal methods and the present proposed method is presented in Table 2. It can be seen that many of the methods used suffered from some significant disadvantages, such as time consumption, the use of corrosive or difficult-to-prepare adsorbent/absorbing materials, operation under non-standard temperature and pressure conditions, and a complicated setup for application.

However, the proposed method is eco-friendly, cost-effective, durable, scalable, and easily processable. The simple proposed compact dry design required minimal manipulation while maintaining good efficiency. The filter material (Nomex) had several advantages such as being heat-resistant, fire-retardant, un-stretchable, non-shrinkable, mechanically stable, chemically inert, and having the ability to remove other gasoline combustion products (e.g., CO, SO_2_, NOx, VOCs, and particulates).

It can be seen that the use of the proposed Nomex/activated carbon sandwich filter system for CO_2_ removal offered clear advantages over many conventional systems such as amine-based scrubbers [34], PSA units [33], and MOFs [44]. It provided a 70 ± 3.4% CO_2_-removal efficiency under ambient conditions. These traditional systems often require high energy input, chemical regeneration, or complex infrastructure. However, the present filtration system involved the use of much lower carbon loading compared with 2.89 mg/cm^2^ for the present dry, compact, and eco-friendly design, which also minimized the environmental impact and made it more suitable for mobile or decentralized applications.

## 4. Conclusions

A simple, compact, and portable filtration system consisting of a sandwich of Nomex fabric containing a thin layer of activated carbon was described for the removal of CO_2_ from the exhaust emissions of gasoline engines. The influence of various experimental parameters was examined and optimized to achieve about a 70 ± 3.4% CO_2_-removal efficiency within 7 min. The proposed solid dry filter system displayed many advantages in terms of simplicity, compact design, scalability, durability, cost-effectiveness, and stability under harsh conditions. Additionally, the filter effectively removed other gaseous combustion products, such as SO_x_, NO_x_, CO, VOCs, and particulates, with 50–95% removal efficiencies. In situ and on-line emission monitoring and data management were conducted through an Internet of Things (IoT)-based gas monitoring system, using mobile internet via the Global System for Mobile Communications (GSM) network and General Packet Radio Service (GPRS) links for secure cloud storage, real-time observation, and remote management. The scalability of the system will permit its application in industrial zones, transportation networks, and urban settings, by adapting the technology to higher-volume filtration and integrating it into existing infrastructures. Its widespread adoption could lead to significant societal benefits, including enhanced public health by reducing respiratory diseases caused by air pollution and contributing to global efforts to meet emission reduction targets under the Paris Agreement.

## Figures and Tables

**Table 1:** Adsorption kinetic parameters for CO_2_ removal using pseudo-first-order and pseudo-second-order models.

Adsorbent	Pseudo-First-Order			Pseudo-Second-Order			
	k_1_ (min^−1^)	*q_e_*_1_ (mg/g)	R^2^	k_2_ (g/(mg·min)	*q_e_*_2_ (mg/g)	*q_e_* ^exp^	R^2^
Nomex/AC filter	0.77519 ± 0.004	1191.5 ± 17.2	0.8207	0.0078 ± 4.2 × 10^−5^	399.6 ± 0.4	400	0.9919

**Table 2:** Comparison of the efficiency of various methods used for the removal of CO_2_ emissions.

Sorbent	Method of Synthesis	Measurement Conditions	CO_2_ Max. Removal Efficiency (%)	Ref.
Ammonia-based scrubber integrated with dual-layer granular bed filter	Engineered assembly (AC + zeolite 5A)	8% aqueous ammonia, room temp., atmospheric pressure	86	[34]
Phthalimide-modified cellulose nanofiber (CNF) aerogel	Surface functionalization of CNF with phthalimide, followed by freeze-drying	85 °C, 95% RH, 1 bar, 8 h	60	[35]
Chlorella sp. NCTU-2 (microalgal photobioreactor)	Isolation and cultivation in porous centric-tube photobioreactor	10% CO_2_ aeration at 0.125 vvm, 26 ± 1 °C, biomass 5.15 g/L	63	[36]
Amine-impregnated silica foam with ultra-large mesopores	Wet impregnation of PEI into silica foam synthesized from sodium silicate under neutral conditions	75 °C, 1 atm dry CO_2_ (80%) for 1 h	80	[37]
Multi-walled carbon nanotube (MWCNT) filter	Deposition of commercial MWCNTs into stainless steel filter housing with Whatman paper	40–85 °C, CNT loading 0.2–1.6 mg/cm^2^, tailpipe gas from mobile sources	60	[38]
[Bmim][DCA] ionic liquid with 20 wt% PAMAM dendrimer	Blending of [bmim][DCA] with 20 wt% PAMAM Gen 0 dendrimer, used in membrane contactor system	Feed gas 14.1% CO_2_, 50–55 °C absorption, 85–90 °C stripping, 1 atm	62	[39]
1- Ethyl -3-methylimidazolium acetate [EMIM^+^] [AC^−^] ionic liquid	Commercial IL dried under vacuum at 85 °C prior to use in packed column	70–80 °C, 4 bar, 7% CO_2_ in N_2_, counter-current flow	70	[40]
Diethanolamine (DEA) aqueous solution in HF membrane contactor	Commercial DEA used in concurrent hollow-fiber module with polypropylene membrane	298 K, 1 atm, 10% CO_2_/N_2_ gas mixture, concurrent shell-tube flow	57	[41]
Lithium oxosilicate (Li_8_ SiO_6_**)**	Solid-state reaction of Li_2_O and SiO_2_, calcined at 800 °C	500–700 °C, 1 atm CO_2_ flow (60 mL/min), isothermal capture	71	[42]
Activated carbon from coconut shell (KOH activated)	Carbonization and chemical activation with KOH, followed by washing and drying	50–100 °C, 0.2–1 bar, CI engine exhaust, 2500 g sorbent	50	[32]
Corona discharge plasma reactor in vehicle muffler	Integration of high-voltage corona discharge system into modified muffler chamber	12 V, 34 Ah battery supply; stationary diesel engine; optimum at 2200 rpm	86	[43]
Unmodified [Cu_3_(btc)_2_]	Standard solvothermal synthesis	25 °C, 18 bar pure CO_2_, gravimetric method	30	[44]
Li-doped [Cu_3_(btc)_2_]	Post-synthetic Li doping using lithium naphthalenide		47	
CNT@[Cu_3_(btc)_2_]	Solvothermal growth on carboxylated MWCNTs		60	
Li@CNT@[Cu_3_(btc)_2_]	Combination of Li doping and MWCNT incorporation		66	
Mesoporous MgO	Vacuum drying and calcination with carbon exotemplate	25 °C, 101 kPa, 99.9% CO_2_	18	[45]
CaO nanopods	CO_2_ bubbling in Ca(OH)_2_ slurry with polymer block, followed by calcination	600 °C, 101 kPa, 60% CO_2_	70	
Nano CaO/Al_2_O_3_	Chemical synthesis from nano CaCO_3_ and aluminum sol	650 °C, 101 kPa, 33.3% CO_2_	60	
MWCNT	Commercial MWCNTs grafted with amine groups	60 °C, 101 kPa, 50% CO_2_	17	
Zeolite (13X) with pressure swing adsorption (PSA)	Commercial zeolite 13X packed into three-bed PSA system	13% CO_2_ in N_2_, 1.5 atm adsorption, 0.05 atm desorption, room temp.	69	[46]
Activated carbon with pressure swing adsorption (PSA)	Commercial BPL activated carbon used in PSA with 3-bed, 7-step cycle	16% CO_2_ in N_2_, 830/760/50 mmHg (adsorption/purge/evacuation), 30 °C	53	[33]
Zeolite 5A with swing adsorption (TSA) and/or vacuum swing adsorption (VSA)	Commercial zeolite 5A packed in stainless steel column; regenerated by heating and/or vacuum	13% CO_2_ in N_2_, adsorption at 25 °C, desorption at 130–210 °C or 6–10 mbar	45	[47]
Zeolite 13X with vacuum swing adsorption (VSA)	Commercial 13X from UOP packed in 3-bed pilot VSA system	12% CO_2_ in dry air, 40–50 °C, 135 kPa adsorption, 5 kPa desorption	70	[48]
Nomex/Activated Carbon Sandwich Filter	Sandwich assembly of Nomex layers with 2.89 mg/cm^2^ AC, heated at 120–130 °C under 520.5 kPa	Gasoline engine exhaust, 10 L/min flow, ambient temp., monitored with IoT system	70 ± 3.4	This work

## Data Availability

The raw data supporting the findings of this study are available from the corresponding authors, S.S.M. Hassan or M.A. Fathy, upon reasonable request.
